# Developing Techniques for the Utilization of Planctomycetes As Producers of Bioactive Molecules

**DOI:** 10.3389/fmicb.2016.01242

**Published:** 2016-08-19

**Authors:** Olga Jeske, Frank Surup, Marcel Ketteniß, Patrick Rast, Birthe Förster, Mareike Jogler, Joachim Wink, Christian Jogler

**Affiliations:** ^1^Department of Microbial Cell Biology and Genetics, Leibniz Institute DSMZ, BraunschweigGermany; ^2^Department of Microbial Drugs, Helmholtz Centre for Infection Research, BraunschweigGermany; ^3^German Centre for Infection Research Association, Partner Site Hannover-Braunschweig, BraunschweigGermany

**Keywords:** Planctomycetes, secondary metabolites, screening, antibiotics, natural products, *Planctopirus limnophila*, *Rhodopirellula baltica*

## Abstract

Planctomycetes are conspicuous, ubiquitous, environmentally important bacteria. They can attach to various surfaces in aquatic habitats and form biofilms. Their unique FtsZ-independent budding cell division mechanism is associated with slow growth and doubling times from 6 h up to 1 month. Despite this putative disadvantage in the struggle to colonize surfaces, Planctomycetes are frequently associated with aquatic phototrophic organisms such as diatoms, cyanobacteria or kelp, whereby Planctomycetes can account for up to 50% of the biofilm-forming bacterial population. Consequently, Planctomycetes were postulated to play an important role in carbon utilization, for example as scavengers after phototrophic blooms. However, given their observed slow growth, such findings are surprising since other faster- growing heterotrophs tend to colonize similar ecological niches. Accordingly, Planctomycetes were suspected to produce antibiotics for habitat protection in response to the attachment on phototrophs. Recently, we demonstrated their genomic potential to produce non-ribosomal peptides, polyketides, bacteriocins, and terpenoids that might have antibiotic activities. In this study, we describe the development of a pipeline that consists of tools and procedures to cultivate Planctomycetes for the production of antimicrobial compounds in a chemically defined medium and a procedure to chemically mimic their interaction with other organisms such as for example cyanobacteria. We evaluated and adjusted screening assays to enable the hunt for planctomycetal antibiotics. As proof of principle, we demonstrate antimicrobial activities of planctomycetal extracts from *Planctopirus limnophila* DSM 3776, *Rhodopirellula baltica* DSM 10527, and the recently isolated strain Pan216. By combining UV/Vis and high resolution mass spectrometry data from high-performance liquid chromatography fractionations with growth inhibition of indicator strains, we were able to assign the antibiotic activity to candidate peaks related to planctomycetal antimicrobial compounds. The MS analysis points toward the production of novel bioactive molecules with novel structures. Consequently, we developed a large scale cultivation procedure to allow future structural elucidation of such compounds. Our findings might have implications for the discovery of novel antibiotics as Planctomycetes represent a yet untapped resource that could be developed by employing the tools and methods described in this study.

## Introduction

Antibiotics are mostly small molecules with antimicrobial activity produced by nearly all sorts of living things. They revolutionized treatment of infectious diseases, saving countless lives. The ability to produce such active molecules – mostly as secondary metabolites – is unevenly distributed among different species (Berdy, 2005). In the kingdom of bacteria, Actinomycetes are the best studied producers, followed by Myxobacteria, Cyanobacteria and certain *Bacillus-* and *Pseudomonas* strains. In total about 13,800 ‘active’ compounds of bacterial origin are known (Berdy, 2005). However, these ‘usual suspects’ in terms of antibiotic production have been heavily screened in the past and the discovery of novel lead structures decreased, while rediscovery rates of known compounds increased (Cooper and Shlaes, 2011). Consequently, only four new classes of antibiotics have been brought to market in the past four decades (Cooper and Shlaes, 2011). This falls far short of demand and only 74 years after the first treatment with ‘natural’ antibiotics, we again face the specter of incurable bacterial infections, now due to multidrug resistant pathogens (Cooper and Shlaes, 2011). To prevent the post-antibiotic era from coming true, the discovery of novel antibiotic structures is key (Fowler et al., 2014). The chances to discover such structures correlate among other factors directly with the phylogenetic distance between the microorganism under study and the already known producers (Müller and Wink, 2014). Thus, phylogenetically distinct bacterial linages might represent a valuable source for novel secondary metabolites. For instance, the candidate phylum ‘Tectomicrobia’ that dwells in association with a marine sponge was recently found to comprise a distinct and promising metabolic repertoire (Wilson et al., 2014). Besides such phylogenetic aspects, most potent antibiotic producers are characterized by large genomes with often more than 8 MB and complex life styles, involving for example differentiation processes (Müller and Wink, 2014). Employing these criteria, entire bacteria phyla could be judged as putative ‘talented’ producers if basic knowledge about their ecology, cell biology and genomic architecture is available.

Thus, we screened the literature with a focus on little studied ‘conspicuous’ bacterial phyla that might represent putative ‘talented’ producers and identified Planctomycetes as candidates. Firstly, these ubiquitous and environmentally important bacteria possess large genomes (Glöckner et al., 2003; Jeske et al., 2013). Secondly, the planctomycetal cell biology is remarkable and was speculated to parallel eukaryotic cells in some aspects (Fuerst and Sagulenko, 2011). While some of these findings, such as the lack of peptidoglycan cell walls have been recently challenged (Jeske et al., 2015; van Teeseling et al., 2015), other traits like their lifestyle switch after cell division, makes them unique among bacteria. Planctomycetes divide mostly through polar budding rather than binary fission, without employing the otherwise universal bacterial division protein FtsZ (Tekniepe et al., 1981; Pilhofer et al., 2008; Lee et al., 2009; Jogler et al., 2012). In the model organism *Planctopirus limnophila* for example, cell division is further coupled to a lifestyle switch, since only sessile stalked mother cells can divide. Flagellated, planktonic daughter cells need to develop into stalked, sessile cells prior to division (Gade et al., 2005; Jogler et al., 2011; Jogler and Jogler, 2013). Attached Planctomycetes can form dense biofilms, preferably on the surface of blooming diatoms (Morris et al., 2006; Pizzetti et al., 2011a) and they represent up to 50% of the bacterial community on *Laminaria hyperborea* (Bengtsson and Øvreås, 2010). Given the slow growth of Planctomycetes under favorable laboratory conditions, some species have doubling times of up to 1 month (Strous et al., 1998), their abundance in such embattled carbon rich habitats in nature, in contrast to the largely oligotrophic surrounding water, appears counterintuitive (Lage and Bondoso, 2014). Most other heterotrophs that dwell in such ecological niches divide much faster (for example 1.2–6.3 h for *Roseobacter* sp., Christie-Oleza et al., 2012) and should outcompete Planctomycetes. However, the phototroph–planctomycetal allelopathic interactions might involve the production of various secondary metabolites that might exhibit antimicrobial activity (defense against other, faster growing, heterotrophic bacteria) or algicidal (to destroy algae, diatoms or cyanobacteria for scavenging). The latter is supported by a positive correlation of planctomycetal abundance with chlorophyll a concentrations, pointing toward remineralization of algal biomass after blooming events through Planctomycetes (Pizzetti et al., 2011b).

Planctomycetes were found to possess secondary metabolite gene clusters employing genome mining, targeting non-ribosomal peptide synthetases (NRPSs) and polyketide synthases (PKSs) encoding genes (Donadio et al., 2007). In the same year, planctomycetal type I polyketide keto-synthase domains were identified in two costal Antarctic sediments (Zhao et al., 2008). Only recently, we followed a holistic approach and combined comprehensive genome mining with physiological studies to improve our understanding of the planctomycetal potential to degrade algal polysaccharides and to produce secondary metabolites (Jeske et al., 2013). We further supported the hypothesis of allelopathic interactions between Planctomycetes and phototrophs and found, from a genomic perspective, Planctomycetes to be ‘talented’ producers.

In this study we employed chemical assays with planctomycetal crude extracts and biological screening methods for live visualization to prove the planctomycetal production of molecules with antibiotic activity. We further developed the entire set of tools ranging from cultivating marine and limnic Planctomycetes in chemical defined media within process-controlled bioreactors, to the preparation of planctomycetal extracts, their chromatographic separation, fractionation and biological screening. Here, we provide the starting point for the in depth investigation of novel planctomycetal antibiotics.

## Materials and Methods

### General Cultivation of Microorganisms

In this study, the novel chemically defined Maintain Medium 1 (MM1) was developed for *P. limnophila* DSM 3776. MM1 consists of artificial freshwater (10 μM NH_4_Cl, 10 μM KH_2_PO_4_, 100 μM KNO_3_, 200 μM MgSO_4_ 7 H_2_O, 100 μM CaCl_2_ 2 H_2_O, 250 μM CaCO_3_, and 300 μM NaHCO_3_), that was supplemented with 20 ml/l mineral salts solution and 5 ml/l vitamin solution (double concentrated) and buffered with either 10 mM HEPES or 100 mM sodium phosphate buffer at pH 7.2. While HEPES was added before autoclaving, the sodium phosphate buffer ingredients (200 mM NaH_2_PO_4_ H_2_O; 200 mM Na_2_HPO_4_ 7 H_2_O) were separately autoclaved and added afterward (28 ml/l of mono salt solution and 72 ml/l di-salt solution). Mineral salt solution and vitamin solution were prepared according to DSMZ medium 621^1^. The metal salts for the mineral salt solution consisted of 250 mg/l Na-EDTA, 1095 mg/l ZnSO_4_ 7 H_2_O, 500 mg/l FeSO_4_ 7 H_2_O, 154 mg/l MnSO_4_ H_2_O, 39.5 mg/l CuSO_4_ 7 H_2_O, 20.3 mg/l CoCl_2_ 6 H_2_O, 17.7 mg/l Na_2_B_4_O_7_ 10 H_2_O of which 50 ml were added per liter of mineral salt solution. To allow growth in MM1, 10 ml/l sterile filtered carbon source (2.5% glucose solution or 5% *N*-acetyl-D-glucosamine (NAG) solution) was added.

For *Rhodopirellula baltica* SH1 DSM 10527, the chemically defined Maintain Medium 2 (MM2) was developed in this study. MM2 is composed of 250 ml/l 2x artificial sea water (46.94 g/l NaCl, 7.84 g/l Na_2_SO_4_, 21.28 g/l MgCl_2_ 6 H_2_O, 2.86 g/l CaCl_2_ 2 H_2_O, 0.384 g/l NaHCO_3_, 1.384 g/l KCL, 0.192 g/l KBr, 0.052 g/l H_3_BO_3_, 0.08 g/l SrCl_2_ 6 H_2_O, and 0.006 g/l NaF) supplemented with 5 ml/l 1 M Tris/HCl pH 7.5, 20 and 5 ml/l vitamin solution (double concentrated, added after autoclaving) while buffered at pH 7.5. To allow growth in MM2, 40 ml/l sterile filtered carbon source (2.5% glucose solution or 5% NAG) was added.

For strain Pan216 the novel M1H NAG ASW culture broth was developed in this study. It is composed of 250 ml/l 2x artificial sea water (46.94 g/l NaCl, 7.84 g/l Na_2_SO_4_, 21.28 g/l MgCl_2_ 6 H_2_O, 2.86 g/l CaCl_2_ 2 H_2_O, 0.384 g/l NaHCO_3_, 1.384 g/l KCl, 0.192 g/l KBr, 0.052 g/l H_3_BO_3_, 0.08 g/l SrCl_2_ 6 H_2_O, and 0.006 g/l NaF), supplemented with 0.25 g/l peptone, 0.25 g/l yeast extract, 20 ml/l mineral salts solution, 20 ml/l 5% (w/v) NAG solution, 10 ml/l 2.5% (w/v) glucose and 5 ml/l vitamin solution (double concentrated), 1 ml/l trace element solution [1500 mg/l N(CH_2_COONa)_3_ H_2_O, 500 mg/l MnSO_4_ H_2_O, 100 mg/l FeSO_4_ 7H_2_O, 100 mg/l Co(NO_3_)_2_ 6H_2_O, 100 mg/l ZnCl_2_, 50 mg/l NiCl_2_ 6H_2_O, 50 mg/l H_2_SeO_3_, 10 mg/l CuSO_4_ 5H_2_O, 10 mg/l AlK(SO_4_)_2_ 12H_2_O, 10 mg/l H_3_Bo_3_, 10 mg/l NaMoO_4_ 2H_2_O, 10 mg/l Na_2_WO_4_ 2H_2_O] and buffered with 10 mM HEPES at pH 8.0.

All strains were cultivated at 28°C in their respective culture broth with slight agitation (80 rpm).

Indicator strains were cultured according to DSMZ cultivation recommendations^2^.

### Phylogeny of Strain Pan216

Pan216 was identified by 16S rRNA gene sequencing after amplification with the modified universal primers 8f (5′-AGA GTT TGA TCM TGG CTC AG-3′) and 1492r (5′-GGY TAC CTT GTT ACG ACT T-3′; Lane, 1991). PCR reactions were performed directly on single colonies or liquid cultures using the *Taq* DNA Polymerase (Qiagen) with one reaction of 25 μl containing 11 μl PCR–grade H_2_O, 2.5 μl 10x CoralLoad buffer, 2.5 μl Q-Solution, 0.5 μl dNTPs (10 mM each), 1 μl sterile bovine serum albumin solution (20 mg/ml), 0.5 μl MgCl_2_ solution (25 mM), 0.125 μl *Taq*-Polymerase (1 U/μl) and 1 μl of each primer (10 pmol). The employed protocol consisted of two steps, the first step with an initial denaturation at 94°C, 5 min, 10 cycles of denaturation at 94°C, 30 s, annealing at 59°C, 30 s, elongation at 72°C, 1 min, followed by the second step with 20 cycles denaturation at 94°C, 30 s, annealing at 54°C, 30 s, elongation at 72°C, 1 min, and a final elongation step at 72°C, 7 min. All PCRs were carried out in an Applied Biosystems^®^ Veriti^®^ thermal cycler (Thermo Fisher Scientific) and PCR products were stored at 4°C until Sanger-sequencing.

Near full length 16S sequences were generated by assembly of the resulting sequences with the ContigExpress application of the Vector NTI^®^ Advance 10 software (Thermo Fisher Scientific).

Alignments of near full length 16S rRNA sequences were performed using the SINA web aligner (Pruesse et al., 2012), corrected manually and used for phylogenetic tree reconstruction (**Supplementary Figure S1**). Tree reconstruction was performed with the ARB software package (Ludwig et al., 2004) using the Maximum likelihood RAxML module and rate distribution model GTR GAMMA running the rapid bootstrap analysis algorithm, the Neighbor Joining tool with Felsenstein correction for DNA and Maximum Parsimony method employing the Phylip DNAPARS module. Bootstrap values for all three methods were computed with 1,000 resamplings including the *Escherichia coli* 16S rRNA gene positions 101-1438. The analysis involved a total of 37 nucleotide sequences of described type strains and outgroup strains (gray box; compare Supplementary Table S2).

### Growth Measurement of Planctomycetes

**FIGURE 1:**
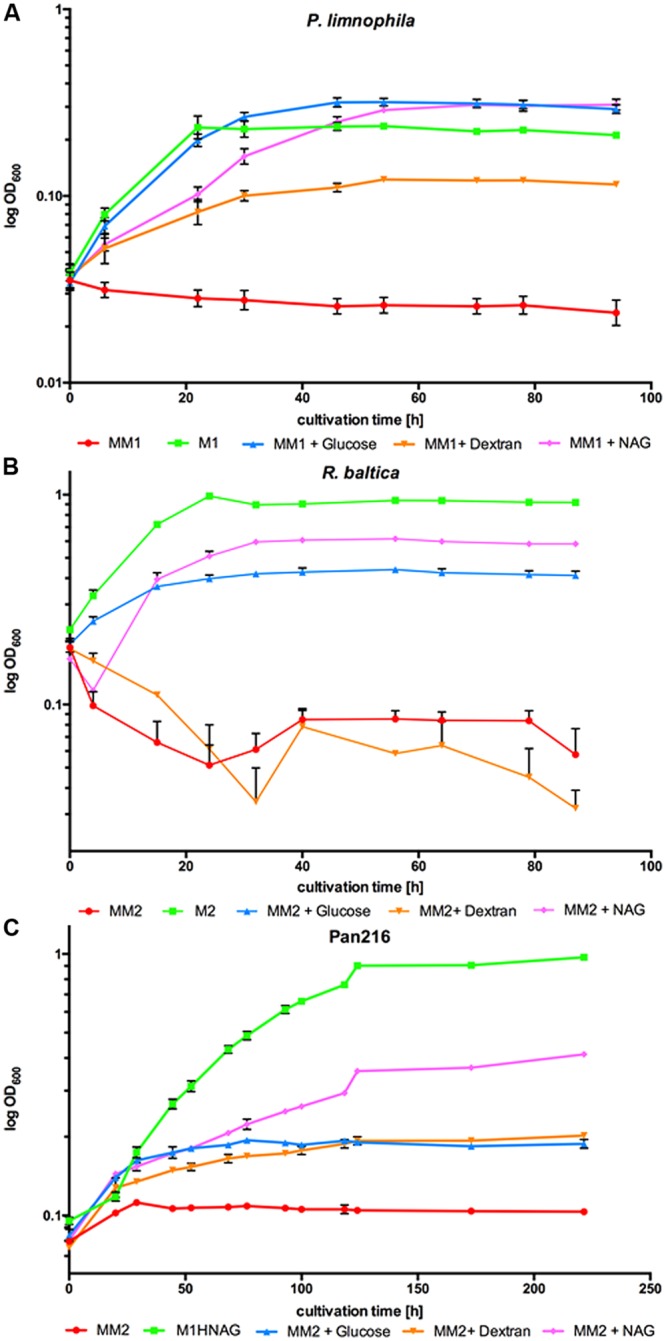
**Growth of *Planctopirus limnophila, Rhodopirellula baltica* and strain Pan216 under different cultivation conditions.** Logarithmic OD_600_ values as measure of growth over 96 h for **(A)** *P. limnophila*, **(B)** *R. baltica* and 255 h for **(C)** strain Pan216 under different cultivation conditions: M1 and M2: complex culture media containing yeast extract, peptone and glucose (green curves). Maintain medium 1 or Maintain medium 2 (MM1 and MM2) are chemically defined but lack a carbon source and thus, unsupplemented, do not allow for bacterial growth of Planctomycetes (red curves). MM1 and MM2 were supplemented with glucose (blue curves), dextran (orange curves) or *N*- acetyl-D-glucosamine (NAG; pink curves) to allow growth of *P. limnophila*, *R. baltica* and strain Pan216. Each time point represents the mean of triplicate measurements.

To measure growth of *P. limnophila* in the different cultivation media (**Figure 1A**), 250 ml baﬄed flasks with 70 ml respective growth medium were inoculated with 10 ml pre-culture, which were washed twice with MM1 prior inoculation. Likewise, *R. baltica* and Pan216 were incubated with different carbon sources (**Figures 1B,C**) and inoculated with a pre-culture washed twice with MM2. Afterward, the respective carbon source was supplemented (4 ml of 2.5% glucose, 4 ml of 2.5% dextran, or 2 ml of 5% NAG). As control *P. limnophila* was grown in M1 and *R. baltica* in M2 medium (Jeske et al., 2013) while Pan216 was grown in M1H NAG ASW medium. Individual growth conditions were analyzed in three biological replicates. The optical density of the culture was measured every 8 and 16 h at 600 nm with a spectrophotometer.

### Cultivation of Planctomycetes in a Bioreactor

The respective growth medium was autoclaved in a 368 l chamber autoclave (BeliMed) within the 10 l culture vessel of the bioreactor (UniVessel, Sartorius). After sterile supplementation of the respective vitamins and carbon source the reactor was incubated for 2 days to exclude contaminations. For sterile inoculation, a planctomycetal pre-culture (0.1 volume) was transferred via a culture bottle into the 10 l culture vessel of the BIOSTAT^®^B system.

*Planctopirus limnophila* was cultured at 28°C in MM1 supplemented with glucose with a constant pH of 7.2 at 100 rpm agitation and a constant air supply (0.5 bar, 0.85 l/min). Samples for glucose consumption, OD_600_ and biomass determination were taken every 24 h (**Figure 3A**).

In contrast, *R. baltica* was cultured in MM2 supplemented with glucose at pH 7.5 with otherwise identical parameters (**Figure 3B**).

For fed-batch cultivation the *P. limnophila* culture was spiked after 7 days with 25 ml NAG and 10 ml vitamin solution and cultured for 10 more days (**Supplementary Figure S3**).

### Extraction of Secondary Metabolites from Planctomycetes

For crude extract preparation, 600 ml of a *P. limnophila* culture were incubated with 2% (v/v) of purified adsorbent resin XAD-16N (Rohm and Haas) at 28°C with 80 rpm agitation in a baﬄed flask for 3 days. The XAD-16N was collected by filtration through an analysis sieve and transferred into an extraction flask. 200 ml acetone were added and incubated in darkness for 1 h at room temperature. To remove the XAD resin the eluate was filtered (Macherey and Nagel 615 ¼, pore size 4–12 μm) after incubation. In parallel, cells were harvested by centrifugation and suspended in 200 ml acetone for 1 h in darkness at room temperature, followed by filtration. Employing a rotary evaporator (Hei VAP-Precision Heidolph) the acetone was removed to yield a solid residue (36°C; at 130 rpm; 1 mbar). The obtained residues were dissolved in 1 ml methanol (pA) and stored at -20°C. Extracts for *R. baltica* and Pan216 were prepared in the same manner. Cultures of Pan216 and XAD were harvested after 5 days of incubation due to their slower growth.

### Measurement of Biomass Production and Glucose Consumption

To determine the dry weight of the planctomycetal cells at the given time points, 25 ml of the respective culture were filtered through two superimposed filters with a pore size of 2.5 μm each (Whatman Grade 5 Qualitative Filter Paper) employing a vacuum pump operating at 800 mbar. The filters were subsequently dried for 60 min at 80°C. Comparison of filter weight before and after filtration gave the bacterial dry mass. The glucose concentration was determined with a test strip (MQuantTM) according to the manufacturer’s description.

### Minimum Inhibitory Concentration Assay

To examine the minimum inhibitory concentrations (MICs) of planctomycetal crude extracts, 26–56 μl of indicator bacteria (for detailed information see Supplementary Table S1) were added to 20 ml of their respective culture medium and mixed well. The indicator strain stocks contained each 1 ml bacteria suspension with an OD_600_ of 3.54 for *E. coli* DSM 1116, 4.05 for *E. coli* TolC, 7.7 *Micrococcus luteus* DSM 1790, 3.69 for *Bacillus subtilis* DSM 10 and 3.86 for *Staphylococcus aureus* DSM 346. Subsequent aliquots were stored at -80°C until usage. 150 μl of the diluted culture was dispensed into each well of a 96-well microtiter plate (initial OD_600_ for the indicator bacteria was 0.01). An additional 130 μl of diluted indicator culture was added to the first row. 20 μl of the planctomycetal crude extracts were added to the first row. A serial dilution of the extract (1:1) was made by transferring 150 μl from one well to the next, this was done from row A to H. For negative and positive controls either 20 μl methanol or 20 μl of the respective culture medium were added instead of planctomycetal crude extracts. Subsequently, the microtiter plates were incubated for 24 h under constant agitation (150 rpm) at 30 or 37°C. After incubation, the growth of the indicator strains was reviewed visually. No turbidity of the media in a well showed an inhibition of the indicator strain.

### Agar Plate Diffusion Assay

To determine antibiotic activity, filter disks (5 mm diameter, pore size 4–7 μm; Whatman No 597) were coated with 90 μl of planctomycetal crude extracts, while methanol (pA) coated disks served as negative control. The treated disks were placed on soft-agar plates inoculated with *B. subtilis* cells and incubated over night at 28°C. Soft-agar plates consisted of a 10 ml 1.5% LB agar layer covered with a second layer of 10 ml 0.7% LB agar inoculated with 100 μl of a *B. subtilis* DSM10 overnight culture.

### Dual Culture Assay

Dual culture assays were performed on M2 1.5% agar plates with *R. baltica* producer and *E. coli* DSM 1116 as indicator strain. Given the different doubling times of both species, *R. baltica* was inoculated first and incubated for 2 days at 28°C. Afterward, *E. coli* was inoculated on the same plate and the agar dishes were incubated for three more days at 28°C. To investigate if growth-altering effects appear in a distance-dependent manner, respective bacteria were plated at different defined distances from each other (2, 10, and 15 mm respectively).

### HPLC-Analysis of Planctomycetal Extracts

High performance liquid chromatography (HPLC) analysis was performed with a Prominence HPLC (Shimadzu) equipped with a Macherey and Nagel, CC 250/4.6 NUCLEODUR 100-5 C18 column (pore size 100 Å, particle size 5 μm) coupled to a SPD-M20A photo diode array (PDA) detector (Shimadzu). The PDA detector recorded all emitted wavelengths, while the wavelength with the highest signal intensities (254 nm) was used for further discrimination of the extracts. 15 μl of the investigated extracts were injected and eluted with a modified methanol-water gradient [solvent A: H_2_O; solvent B: methanol, gradient: 5% B for 10 min increasing to 100% B in 15 min, maintaining 100% B for 30 min, flow rate (FR) 0.6 ml/min]. Prior to the analysis of the extracts, the column was equilibrated with pure methanol. Results were analyzed using the LCsolution Shimadzu Software.

### HPLC Fractionation

An Agilent 1260 Series HPLC-UV system equipped with a Waters, XBridge BEH C18, 2.1 mm × 100 mm column (pore size 135 Å, particle size 3.5 μm) was used for the chromatographic fractionation of crude extracts. The same HPLC gradient was used as for the high-resolution electrospray ionization mass spectrometry (HRESIMS) instrument (described below). The flow through was collected in 30 s intervals into a 96-well microtiter plate. Afterward, the plates were dried by a constant nitrogen-flush for 40 min, inoculated with 150 μl indicator bacteria per well and incubated as described above. After 24 h the plates were evaluated and documented employing a custom-made mirror stand and a CANON EOS 10D digital camera.

### Mass Spectra

Electrospray ionization mass spectrometry (ESIMS) spectra in positive and negative mode were obtained with an Agilent 1260 Infinity Series HPLC-UV system (column 2.1 mm × 50 mm, 1.7 μm, C18 Acquity UPLC BEH (Waters), solvent A: H_2_O + 0.1% formic acid; solvent B: ACN + 0.1% formic acid, gradient: 5% B for 0.5 min increasing to 100% B in 19.5 min, maintaining 100% B for 5 min, FR 0.6 ml/min, UV detection 200–600 nm) equipped with a diode array detector and an ESIiontrap MS detector (Amazon, Bruker).

High-resolution electrospray ionization mass spectrometry spectra were obtained with an Agilent 1200 series HPLC-UV system [solvent A: H_2_O/ACN (95/5) + 5 mM/l of NH_4_Ac + 40 μl/l of acetic acid; solvent B: H_2_O/ACN (5/95) + 5 mM/l of NH_4_Ac + 40 μl/l of acetic acid, gradient: 10% B increasing to 100% B in 30 min, maintained 100% B for 10 min; FR = 0.3 ml/min; UV detection 200–600 nm] combined with an ESI-TOF-MS (Maxis, Bruker; scan range 100–2500 m/z, rate 2 Hz, capillary voltage 4500 V, dry temperature 200°C).

## Results

### Chemically Defined Growth Conditions for Marine and Limnic Planctomycetes

We selected the marine species *R. baltica* SH1 DSM 10527 and the limnic organism *P. limnophila* DSM 3776 as model Planctomycetes together with the phylogenetically distant and recently isolated strain Pan216 (**Supplementary Figure S1**) for subsequent experiments. For both established model species, optimized growth conditions in complex media, containing rather high amounts of yeast extract and peptone, have been previously described (Jogler et al., 2011; Jeske et al., 2013). The optimal growth conditions in rich media for strain Pan216 were determined in this study (see Materials and Methods).

To enable ecomimetic experiments that mimic certain natural conditions in the laboratory, and to optimize HPLC detection and fractionation, we developed a chemically defined and carbon source free maintain medium for limnic- (MM1) and marine conditions (MM2). *P. limnophila*, *R. baltica*, and Pan216 cells survived under such conditions, but were not able to divide and grow without supplementation of any maintain medium with a carbon source (**Figure 1**, red curves). After a starvation period (5–9 days) in the respective Maintain Media, all three strains were able to fully recover when specific carbon and nitrogen sources were added. Remarkably, *P. limnophila* grew even more efficiently in MM1 + glucose (same growth rate but higher biomass attained; **Figure 1A**, blue curve) or MM1 + NAG (lower growth rate but higher biomass attained; **Figure 1A**, pink curve), than in the originally described isolation medium (**Figure 1A**, green curve). *R. baltica* grew less efficiently with MM2 + glucose (**Figure 1B**, blue curve) or MM2 + NAG (**Figure 1B**, pink curve) and did not reach the same OD_600_ compared to the medium culture broth M2 (**Figure 1B**, green curve). Pan216 grew best if fed with M1H NAG ASW medium developed in this study (**Figure 1C**, green curve). The chemically defined MM media were also supplemented with other carbon sources such as the complex polysaccharide dextran (**Figure 1**, orange curves) that provided growth for *P. limnophila* and Pan216 but not for *R. baltica.* In the case of *R. baltica*, the supplementation of NAG (**Figure 1B**, pink curve) led to initial biofilm formation resulting in an early decrease of the cultures optical density. This effect was even stronger after adding dextran (**Figure 1B**, orange curve) and explains why the optical density was lower than in the control culture (**Figure 1B**, red curve) without any carbon source added. Thus certain carbon sources might trigger a lifestyle switch from planktonic growth to biofilm formation.

The adsorber resin XAD binds unspecifically to both secreted secondary metabolites and components of the complex growth media M1 and M2, resulting in visible media component signals in HPLC measurements even when no-bacteria medium control samples were measured (**Supplementary Figures S2A,C**). However, extraction of our newly developed MM1 and MM2 media with XAD showed no media component signals in HPLC analysis (**Supplementary Figures S2B,C**), making them the preferred choice to investigate the influence of trigger substances on secondary metabolite production in Planctomycetes.

### Ecomimetic Cultivation of Planctomycetes

**FIGURE 2:**
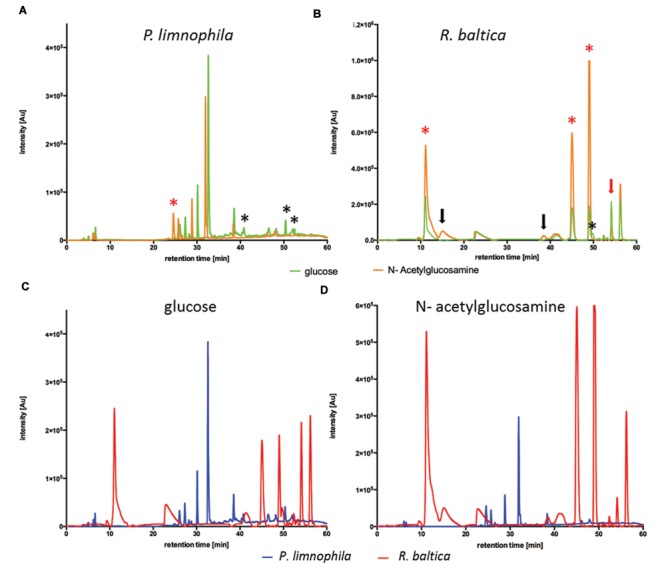
**High performance liquid chromatography (HPLC) analysis of *R. baltica* and *P. limnophila* extracts. (A)** HPLC-analysis of extracts generated from *P. limnophila* cultivated in MM1 supplemented with glucose (green curve) and NAG (orange curve). Feeding glucose resulted in higher major peaks than NAG. Three minor peaks were exclusively produced if glucose was added (black asterisk), while one major peak was unique to NAG cultivation (red asterisk). **(B)** HPLC-analysis of extracts generated from *R. baltica* cultivated in MM2 supplemented with glucose (green curve) or NAG (orange curve). The intensity of the secondary metabolite peaks was higher in extracts from *R. baltica* cultivated with NAG as carbon source compared to extracts from cultivation with glucose. Extracts from glucose cultivation showed five peaks after 12 and 40–56 min with intensity only up to 2.2 × 10^5^ Au, while same signal peaks from extracts from NAG cultivation showed intensity up to 10^6^ Au. Three of the major peaks were significantly higher (red asterisk), while one showed less signal intensity (red arrow). The HPLC spectrum contains two additional minor peaks (black arrows) while one minor peak vanished (black asterisk). **(C)** Comparison of HPLC- chromatograms of XAD extracts from *P. limnophila* cultivated in MM1 (blue curve) and *R. baltica* in MM2 (red curve) supplemented with glucose as sole carbon source. Secondary metabolites signals from *P. limnophila* extracts were detected after a retention time of 7 and 26–38 min with an intensity of 5 × 10^4^ up to 3.8 × 10^5^ Au, while for *R. baltica* a signal was detected after 10 min with an intensity of 2.5 × 10^5^ Au and additional signals were detected after 44–57 min with an intensity of 1.8 × 10^5^ up to 2.4 × 10^5^ Au. **(D)** Comparison of HPLC- chromatograms of XAD extracts from *P. limnophila* cultivated in MM1 (blue curve) and *R. baltica* in MM2 (red curve) supplemented with NAG as sole carbon source. Secondary metabolite signals from *P. limnophila* were detected with intensity up to 3 × 10^5^ Au after a retention time of 25–38 min while secondary metabolite peaks from *R. baltica* extracts after 12 and 40–56 min with intensity up to 10^6^ Au.

The chemically defined Maintain Media (MM) were developed to enable ecomimetic experiments which attempt to recreate environmental situations under defined conditions in the laboratory. The rational was that certain environmental cues, such as a particular carbon source, would stimulate secondary metabolite production in Planctomycetes, and consequently alter the peak profile in subsequent HPLC analysis. For a proof of principle, we simulated the planctomycetal interaction with cyanobacteria by adding a key component of bacterial cell walls –NAG – to our MM. Since cyanobacteria occur in both, marine- and limnic habitats, we performed the evaluation of our MMs with *R. baltica* (marine) and *P. limnophila* (limnic) in MM2 and MM1 respectively. After cultivation with XAD for 3 days at 28°C, XAD extracts were analyzed *via* HPLC. For *P. limnophila*, three minor peaks were exclusively detected when cells were cultivated with glucose as sole carbon source (**Figure 2A**, black asterisk), while one peak was unique to cultivation with NAG as sole carbon source (**Figure 2A**, red asterisk). When cultivated with glucose, *R. baltica* produced secondary metabolites that led to five major and five minor peaks in the HPLC chromatogram (**Figure 2B**, green curve). In contrast, when fed with NAG, the subsequent HPLC chromatogram contains two additional minor peaks (**Figure 2B**, black arrows) while one minor peak vanished (**Figure 2B**, black asterisk). Three of the major peaks were significantly higher (**Figure 2B**, red asterisk), while one showed less signal intensity (**Figure 2B**, red arrow). The overall signal intensity of *P. limnophila* extracts showed less variation between the cultivation conditions than *R. baltica* did. However, comparison of extracts from *P. limnophila* (**Figures 2C,D**, blue) and *R. baltica* (**Figures 2C,D**, red) showed that both organisms display entirely different HPLC peak patterns.

Thus our Maintain Media allow ecomimetic experiments that lead to the production of different metabolites in response to different carbon sources.

### Screening of Planctomycetal Extracts for Antimicrobial Activity

One aim of this study was to develop cultivation techniques that could stimulate the production of bioactive molecules with antimicrobial activity as demonstrated in the previous section. However, extracts obtained in such ecomimetic experiments require a screening procedure to determine potential antimicrobial activity. Thus, for the first time we demonstrate different screening methods for planctomycetal extracts and strains and evaluated their usefulness.

The semi-quantitative agar plate diffusion assay was used to determine the inhibitory effect of *R. baltica* cell- and XAD resin extracts obtained from *R. baltica* cultures against *B. subtilis*. While the negative control and the cell extract disks showed no influence on bacterial growth, the planctomycetal XAD extract displayed a zone of *B. subtilis* growth inhibition, pointing toward an antibiotic molecule produced by *R. baltica* (**Figure 4A**).

The dual culture assay revealed that reducing the distance between *R. baltica* and *E. coli* K12 increased the growth inhibition effect (**Figure 4B**). *E. coli* grew to highest density with the maximum chosen distance of 15 mm. When the distance to *R. baltica* was reduced to 10 mm, the *E. coli* grew less well. Ultimately, only minor growth of *E. coli* was observed when the distance was reduced to 2 mm distance.

The MIC assay was performed for *R. baltica* extracts (**Figure 4C**). Several dilution series were prepared against *E. coli* TolC, *S. aureus* and *M. luteus*. Only the highest concentration of *R. baltica* XAD extract in the first well showed an effect against the *E. coli* TolC strain (**Figure 4C**: XAD). However, a strong inhibitory effect against *S. aureus* was observed with the *R. baltica* XAD extract (**Figure 4C**: XAD). The first three wells of the dilution series showed a distinct inhibition of growth. The lowest concentration in which the bacterial growth was inhibited therefore contained the antibiotic at MIC. In contrast, *S. aureus* treated with the cell extract grew normally and showed no signs of growth inhibition (**Figure 4C**: cell).

Likewise, only the XAD extract had a strong inhibitory effect against *M. luteus* in the first four wells (**Figure 4C**: XAD), whereas the cell extract had no effect on *M. luteus* (**Figure 4C**: cell). However, since no bacterial growth could be detected in the first two wells of the dilution series for the negative control, the effect of the first two wells in the XAD column might be caused by methanol rather than by a potential antibiotic within the crude extract. However, bacteria were not inhibited in the third well of the negative control, so extracts with activity at this dilution could still contain an active compound.

Thus, all three evaluated screening methods, agar plate diffusion, dual culture, and MIC assay were useful to analyze planctomycetal antimicrobial activity. For the first time we demonstrated antibacterial activity of *R. baltica* against both, Gram-negative and Gram-positive bacteria.

### Correlation of Antimicrobial Activity with Distinct HPLC Peaks

After development of ecomimetic cultivation, biological screening methods, and protocols to facilitate the analysis of planctomycetal antibiotic production, next the HPLC signals need to be correlated with biological activity to facilitate structure elucidation of the antibiotic molecules. Thus, we deve loped a procedure that allows correlation of HPLC peaks with antimicrobial activity of planctomycetal extracts. Again, a limnic (*P. limnophila*) and a marine species (strain Pan216) were chosen for the proof of principle study. After cultivation and extraction of cultures with adsorbent resin, the MIC assay screening against indicator strains (Supplementary Table S1) was performed. Once the analyzed extract showed an activity against one of the indicator strains (**Figures 4C** and **5**), a semi preparative HPLC run was performed (**Figure 5**). Our integrated approach consists of utilizing HPLC instruments coupled with both HRESIMS as well as a fractionation collector for 96-well microtiter plates, which are used in a subsequent bioassay against the prior determined strains. Both HPLC systems were equipped equally, so that chromatograms and retention times can directly be compared. Thereby it is possible to assign the activity observed for the crude extract to individual peaks, and simultaneously to determine their molecular formula (**Figure 5**; **Table 1**). Furthermore, tandem mass spectrometry (MS/MS) and UV/Vis spectra can sometime provide early information about the chemical structure of the desired substance. Although, the HPLC gradient and solvents can be varied, the use of a water/acetonitrile (ACN) system provided best chromatographic separation and we suggest this as standard procedure for the analysis of planctomycetal extracts. Since the use of formic acid did not lead to the reproducible detection of bioactive wells, we concluded that some of the bioactive compounds might be degraded under acidic conditions and consequently we used an acetate buffered solvent system instead.

**Table 1:** Antibacterial metabolites found in *P. limnophila* and Pan216.

Organism	Rt [min]	*m/z*^a^	Molecular formula	Hits in DNP	DBE	MS/MS	Possible fragments
*P. limnophila* R2	22.4	**474.3880** 947.7514 969.7333	C_26_H_57_NO_6_ C_52_H_103_N_2_O_12_ C_52_H_102_N_2_O_12_Na	0	2	456.3685 317.2845 236.1495 217.1954	C_26_H_50_NO_5_ C_22_H_37_O C_10_H_22_NO_5_ C_16_H_25_
Pan216 R1	27.6	**331.2642** 353.2461	C_22_H_35_O_2_ C_22_H_34_O_2_Na	132	6	313.2532 295.2429 271.2429 247.1703 233.1551 217.1954 203.1792 187.1119 177.1638	C_20_H_34_ONa C_20_H_32_Na C_18_H_30_ONa C_14_H_24_O_2_Na C_13_H_22_O_2_Na C_14_H_26_Na C_13_H_24_Na C_11_H_16_ONa C_11_H_22_Na
	25.8	329.2481	C_22_H_33_O_2_	38	7	/	/
	25.1	301.2143	C_18_H_30_O_2_Na	61	4	/	/
Pan216 R2	21.8	**294.0532**	C_12_H_12_N_3_O_4_S	0	8	259.0844 230.0451	? C_12_H_8_NO_4_

*^a^Bold mass was further processed by MS/MS.*

Two replicate cultivation and extraction experiments were performed for both *P. limnophila* and strain Pan216.

Fractionation of *P. limnophila* replicate R1 resulted in inhibition of various wells. The obtained results were not reproducible and no activity could be assigned to peaks in the UV chromatogram.

In contrast, repeated fractionation of 15 μl extract each for *P. limnophila* replicate R2 led to reproducible inhibition of *B. subtilis* growth in well ‘B6’, which had been collected from 23 to 23.5 min (**Table 1**). The chromatogram showed a major peak at this retention time (**Figure 5A**). The same peak was observed at a retention time of 22.4 min in the HRESIMS system. Its molecular formula C_26_H_51_NO_6_ was deduced from its [M+H]^+^ peak at *m/z* 474.3880 as well as its [M+Na]^+^, [2M+H]^+^, and [2M+Na]^+^ peaks at *m/z* 496.3608, 947.7514, and 969.7325, respectively (**Table 1**). The assignment of the molecular ion cluster was further confirmed by the observation of the [M+CO_2_H]^-^ peak at *m/z* 518.3 in the ESIMS spectrum measured in negative mode. A search with the molecular formula C_26_H_51_NO_6_ in the Dictionary of Natural Products (DNPs) on DVD resulted in no known metabolites (**Table 1**).

In the case of strain Pan216, both replicates resulted in different inhibition patterns as well. For replicate R1 inhibition of wells B7, C7, and G7 was observed that correspond to three major peaks of the slightly lipophilic region of the chromatogram (**Figure 5B**). These three peaks were detected in HRESIMS spectrum at 25.1, 25.8, and 27.6 min and molecular formulae of C_18_H_30_O_2_, C_22_H_32_O_2_, and C_22_H_34_O_2_ were assigned from their [M+H]^+^ and [M+Na]^+^ peak pairs (**Table 1**). These assignments were further confirmed by [M+H]^+^ [M-H]^-^ pairs in positive/negative mode ESIMS spectra. The search for these molecular formulae in the DNP resulted in 122, 38, and 61 hits (**Table 1**).

Neither of those peaks were observed in the chromatogram of Pan216 replicate R2 nor was activity observed in wells B7-G7. Instead, well C6 (23.5–24 min) was constantly inhibited. The corresponding peak shows characteristic UV absorption maxima at 244 and 294 nm, which might suggest an aromatic residue. The [M+H]^+^ peak in the HRESIMS spectrum at *m/z* 294.0532 could indicate the molecular formula as C_12_H_11_N_3_O_4_S, which would explain the observed +2 satellite in the molecular ion cluster as the ^34^S isotope. However, the other possibilities for the molecular formula C_13_H_12_NO_5_P and C_20_H_7_NO_2_ cannot be excluded. In the ESIMS spectra in negative mode [M-H]^-^ respectively [2M-H]^-^ peaks at 291.1 and 585.0 confirm the assignment. There is no known natural product included in the DNP for any of the three molecular formulae.

Thus we demonstrated a procedure to correlate HPLC peaks of planctomycetal extracts with antimicrobial activity that allows subsequent purification of the compound of interest. Initial data pointed towards novel bioactive molecules with antimicrobial activity produced by Planctomycetes.

### Cultivation of Planctomycetes in Process Controlled Bioreactors

Thus far we have presented tools and procedures for the discovery of planctomycetal bioactive molecules, yet one important feature is missing if they are to be useful: large scale cultivation. Anammox Planctomycetes are routinely cultivated in different types of bioreactors in small, large and industry scale, mainly for waste water treatment (for review see, van Niftrik and Jetten, 2012). However, little to no experience exists with the planctomycetal orders Phycisphaerales and Planctomycetales. The known species belonging to the order Phycisphaerales have small genomes with few secondary metabolite related clusters while species of the order Planctomycetales were found to comprise large genomes with many secondary metabolite related genes (Jeske et al., 2013). Among Planctomycetales, only the cultivation of *R. baltica* in a small-scale custom-made chemostat was described thus far (Frank et al., 2011). However, such experiments were not performed with the intention to obtain secondary metabolites.

**FIGURE 3:**
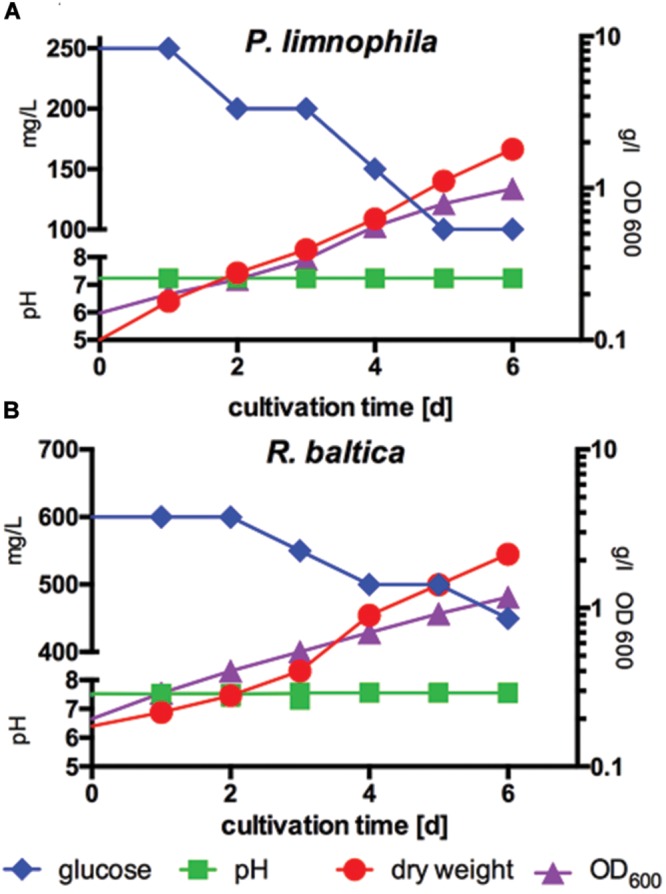
**Measurement of biomass production compared to optical density and glucose consumption of *P. limnophila* and *R. baltica* at constant pH. (A)** Biomass production of *P. limnophila* compared to optical density at 600 nm (OD_600_) and glucose consumption at constant pH over 6 days in MM1 supplemented with glucose in a 10 l fermenter. Glucose concentration decreased from 250 to 100 mg/l (blue curve), while the OD_600_ increased from 0.15 up to 0.99 (purple curve) and correlating with the OD_600_ also the dry weight increased from 0.1 g/l up to 1.8 g/l (red curve) during cultivation. The pH was 7.2 (green curve). **(B)** Biomass production of *R. baltica* compared to, OD_600_-value and glucose consumption at constant pH over 6 days in MM2 supplemented with glucose in a 10 l fermenter. Glucose concentration decreased from 600 to 450 mg/l (blue curve), while the OD_600_ increased from 0.2 to 1.170 (purple curve) and correlating with the OD_600_ also the dry weight increased from 0.18 g/l and to 2.18 g/l (red curve) during cultivation. The pH was stabilized at 7.5 (green curve).

In this study, we developed the required media and procedures to cultivate both limnic (*P. limnophila*) and marine (*R. baltica*) Planctomycetes in a 10 l bench-top bioreactor (BIOSTAT^®^B, Sartorius). Our protocols (see Materials and Methods) fit to batch (**Figure 3**) and fed-batch (**Supplementary Figure S3**) cultivation in different sized stirring tank reactors as our reactor system allows seamless scale-up, at least for up to 100 l.

First, we determined optimal growth conditions in maintain medium supplemented with glucose (**Figures 3A,B**) and obtained optimal biomass production, whereby the pH was kept constant at 7.5 for *R. baltica* and at 7.2 for *P. limnophila*. Since HEPES is used routinely to buffer planctomycetal culture media, the future scale-up might be limited by high costs of this chemical. Thus, we explored different buffer systems and found that at least the growth of *P. limnophila* is not affected by phosphate buffering (see Materials and Methods). Growth was measured by OD_600_ and dry weight, while glucose consumption was determined in addition (**Figures 3A,B**). While both curves, dry weight and OD_600_, comprise a similar shape for *R. baltica*, the slope of the OD_600_ curve for *P. limnophila* is steeper than the curve representing dry weight measurements.

Paralleling the extraction procedure employed for flask cultures, we added XAD to the bioreactor that was finally harvested with an analyze sieve prior to extraction. Thus, even for planctomycetal large-scale cultures our procedure allows small volume downstream processes, significantly reducing the amount of organic solvents and effort for the extraction process.

When two independent *P. limnophila* 5 l cultivation batches were employed for XAD extraction and subsequent HPLC analysis, the resulting metabolite profiles differed slightly (**Supplementary Figure S4**). Eight minor peaks (red asterisks) and one major peak (black asterisks) occurred only in one of the two replicates, while only the three major peaks (black arrows) were found in both extracts.

Thus, we developed a large scale cultivation approach for aerobic Planctomycetes that reduces cost and effort but still lacks 100% reproducibility.

## Discussion

The aim of this pilot study was to develop the tools and procedures required to produce, isolate, and analyze secondary metabolites from Planctomycetes. We employed different marine and limnic Planctomycetes as a proof of principal for each step of our pipeline.

The first step was the development of cultivation conditions that support the production of secondary metabolites. To determine a suitable cultivation strategy, we took the ecology of Planctomycetes into account. It has been demonstrated by both us and others that Planctomycetes interact with phototrophs, such as cyanobacteria, in marine and limnic habitats and that the Planctomycetes can even dominate biofilms in these carbon-rich ecological niches (Bengtsson and Øvreås, 2010; Bengtsson et al., 2010, 2012; Pizzetti et al., 2011a,b; Jeske et al., 2013). Due to the slow growth of Planctomycetes, we hypothesized that their dominance in biofilms could be due to production of antibiotics, which could provide an advantage over faster-growing bacteria. To elucidate whether environmental cues can trigger the production of secondary metabolites, we first developed both a limnic (MM1) and a marine (MM2) Maintain Medium (**Figure 1**). This development is vital, because it dramatically reduces the culture-media components in acetone extracts to zero (**Supplementary Figure S2**). Thus, the MM allows for easy detection and characterization of produced metabolites. In addition, changes in production of bioactive molecules caused by altered growth conditions could be easily monitored. Such alterations could, for example, be achieved by supplementing the MM with different carbon sources. Furthermore, ecomimetic experiments can replicate the environmental interactions of Planctomycetes and phototrophs in a chemically defined manner, allowing for determination of environmental cues which are currently unknown. In a proof of principle study we found that NAG, a main component of the cyanobacterial cell wall, altered the secreted secondary metabolome of both *P. limnophila* and *R. baltica* (**Figure 2**). This finding is not surprising, since it is well-known that changes in the composition of cultivation media can affect the biosynthesis of secondary metabolites (Berdy, 2005). In the past, selection of growth media composition to stimulate the expression of ‘silent’ secondary metabolite genes or clusters was rather random. Here, we utilize the chemical characteristics of the planctomycetal ecological niche to guide alterations of the medium composition which led to the production of novel secondary metabolites. Algae and bacteria produce a multitude of different compounds and it has been shown by both our group and others that many of these molecules can be utilized by Planctomycetes (Wegner et al., 2012; Jeske et al., 2013). Thus, our Maintain Medium provides a base medium that allows for addition of many kinds of substrate combinations. Subsequent analysis of changes in the secondary metabolome can be correlated with environmental cues (substrates) present in the media. Furthermore, the addition of XAD adsorber resin allowed for easy and low solvent-volume extraction and analysis of such molecules.

After developing chemically defined growth conditions and ecomimetic, the second step in our pipeline is the screening for biological activity. We successfully evaluated agar plate diffusion, dual culture, and MIC assays and found all three methods very useful to screen Planctomycetes. An initial screen with the cheap and easy dual culture approach allows for determination of potential antibiotic producers, whilst the agar plate diffusion and the MIC assay allow for further purification of extracts and isolation of active components. Most importantly, we found evidence for antimicrobial activity of Planctomycetes against both Gram-negative and Gram-positive bacteria. This finding might support our hypothesis of the planctomycetal interaction with phototrophs.

**FIGURE 4:**
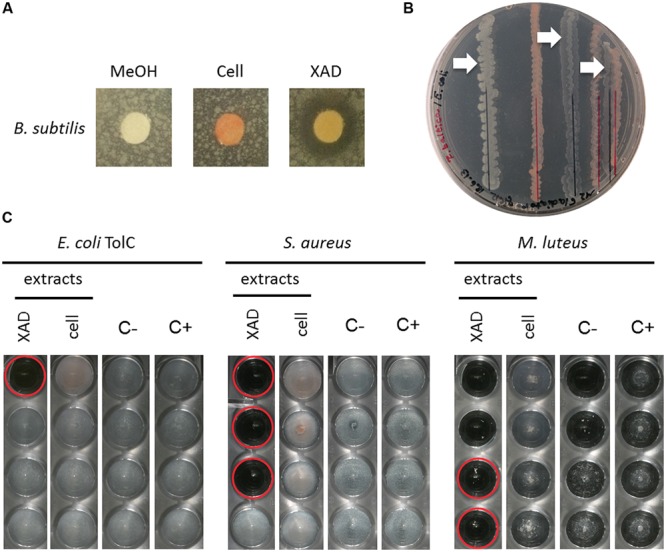
**Activity assays of *R. baltica* methanol extracts. (A)** Agar plate diffusion assay of *R. baltica* methanol cell extracts against *Bacillus subtilis*. Disks are coated with 90 μl methanol (MeOH), *R. baltica* cell or XAD extract respectively and incubated over night on soft-agar plates inoculated with *B. subtilis*. The clear areas around the disks treated with XAD extract show a distinct zone of *B. subtilis* growth inhibition, while methanol or cell extract show no effect. **(B)** Dual culture assay of *R. baltica* and *E. coli*. *E. coli* grew best with the maximal chosen distance of 15 mm to *R. baltica*. *E. coli* cells grew weaker when the distance was reduced to 10 mm and weakest at 2 mm (white arrows). **(C)** Dilution minimum inhibitory concentration (MIC) assay with *R. baltica* cell and XAD extracts. Growth inhibitory effect of cell and XAD methanol extracts on: treated *E. coli* TolC deletion mutant, *S. aureus* DSM 346 and *M. luteus* DSM 1790 cells. XAD: serial dilution column with XAD extract; Cell: serial dilution column with cell extract; C-: serial dilution column with methanol as negative control; C+: respectively untreated cells as positive control. Red circles highlight inhibited cells. If the same methanol concentration also inhibited the growth, the effect of the extracts was not taken into account (see **C** first two wells of XAD extract). The last well in which no bacteria grow contains the antibiotic at its minimal inhibitory concentration.

The third step is the correlation of bioactivity in a crude extract with a particular HPLC peak. This step is crucial for isolation and purification of bioactive compounds, facilitating subsequent structure elucidation. We developed a comprehensive approach by combining analytical and preparative HPLC with direct fractionation of extracts into 96-well plates that can be subsequently employed for activity screening (**Figure 4**). While the elucidation of distinct structures is beyond the scope of this study we gained some insights into the chemical composition of planctomycetal antibiotic molecules. For example, in *P. limnophila* replicate #2, an active metabolite had a molecular formula of C_26_H_51_NO_6_, which is not present in the DNP, suggesting it could be a novel compound. Although *de novo* structure elucidation is impossible with MS/MS data alone, early indications can be obtained by fragmentation ions. For example, fragments of the aforementioned compound indicate that the structure consists of a long aliphatic chain and a heteroatom-containing core structure. Pan216 replicate #2 contained an active metabolite with molecular formula of C_12_H_11_N_3_O_4_S, this was also not in the DNP and suggests a new natural product. UV absorption maxima at 244 and 294 nm might indicate an aromatic or heteroaromatic residue. However, Pan216 replicate #1 showed no evidence of this active metabolite. Three distinct metabolites inhibiting the growth of *B. subtilis* were also produced in Pan216 replicate #2. The active metabolites had molecular formulae of C_22_H_34_O_2_, C_22_H_32_O_2_, and C_18_H_30_O_2_, which corresponds to 6, 7, and 4° of unsaturation, respectively. Dozens of compounds are known for these molecular formulae and so we cannot identify these metabolites nor state that these metabolites are new.

**FIGURE 5:**
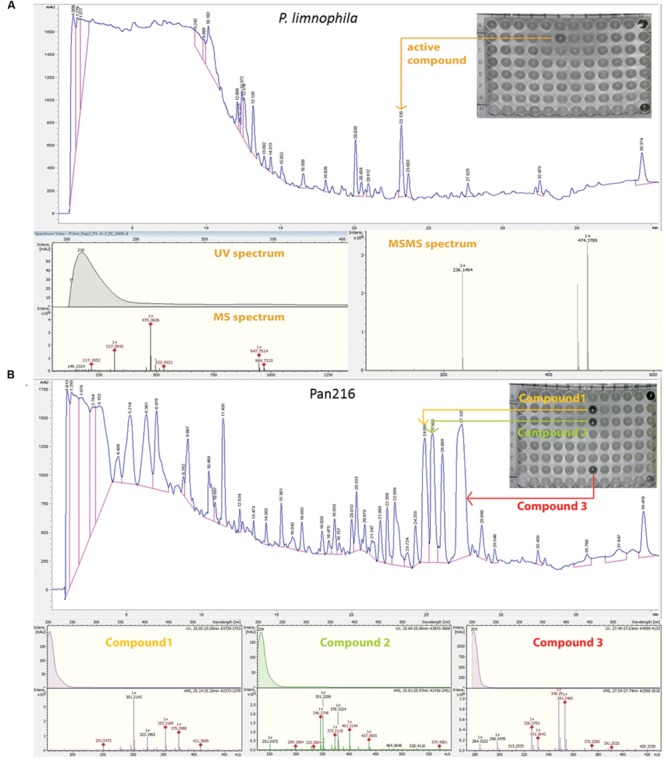
**Fractionation analysis of planctomycetal crude extracts. (A)** Combined chromatogram of HPLC fractionations, UV/Vis, mass- and tandem mass (MS/MS) spectrometry to identify the active compound, which causes the growth inhibition of *P. limnophila* crude extract #2 against *B. subtilis* on a 96 well plate. Fractionation of 15 μL extract for *P. limnophila* replicate #2 led to reproducible inhibition of *B. subtilis* growth in well ‘B6’, which had been collected from 23 to 23.5 min. The chromatogram shows a major peak at this retention time. The same peak was observed at a retention time of 22.4 min in the HRESIMS system. Its molecular formula C_26_H_51_NO_6_ was deduced from its [M+H]^+^ peak at *m/z* 474.3880 as well as its [M+Na]^+^, [2M+H]^+^, and [2M+Na]^+^ peaks at *m/z* 496.3608, 947.7514, and 969.7325, respectively (**Table 1**). The assignment of the molecular ion cluster was further confirmed by the observation of the [M+CO_2_H]^-^ peak at *m/z* 518.3 in the ESIMS spectrum measured in negative mode. **(B)** Combined chromatogram of HPLC fractionations, UV/Vis, mass spectrometry to identify three active compounds, which causes growth inhibition of *B. subtilis* of Pan216 crude extract #1 in a 96 well plate. In case of strain Pan216 both replicates resulted in different inhibition patterns as well. Inhibition of wells B7, C7, and G7 was observed that correspond to three major peaks of the slightly lipophilic region of the chromatogram. These three peaks were observed in HRESIMS spectrum at 25.1, 25.8, and 27.6 min; molecular formulae of C_18_H_30_O_2_, C_22_H_32_O_2_, and C_22_H_34_O_2_ were assigned from their [M+H]^+^ and [M+Na]^+^ peak pairs (**Table 1**). These assignments were further confirmed by [M+H]^+/^ [M-H]^-^ pairs in positive/negative mode ESIMS spectra. Wells A12 and H12 are negative controls treated with tetracycline and crude extract respectively.

Both the metabolome and the bioactivity varied strongly between the replicates of *P. limnophila* and strain Pan216, implying that the production of secondary metabolites is highly variable, responsible factors could include the inoculum, oxygenation, growth stage, formation of aggregates, as well as degradation of compounds during cultivation. Thus, this study revealed that single planctomycetal strains might be able to produce a multitude of antimicrobial secondary metabolites. Therefore, Planctomycetes do not only contain the gene clusters for secondary metabolite production, but actually produce compounds with antibacterial activity. However, structure elucidation of such bioactive molecules requires large scale cultivation of planctomycetal producer strains.

To facilitate future structure elucidation attempts, the last step of our pipeline is the cultivation of marine and limnic Planctomycetes in large scale stirring tank bioreactors. We choose a 5 l volume in 10 l computer controlled bioreactors as this allows for easy scale-up. While establishing the protocols we noticed a disparity between the approximate doubling times determined by OD_600_ (57 h) and dry weight (47 h) for *P. limnophila*, and OD_600_ (62 h) and dry weight (41 h) for *R. baltica*. Therefore, future experiments using dry weight instead of optical density might provide better correlation between growth phase and secondary metabolite production. Whilst HPLC analysis of crude extracts of two replicates of 5 l *P. limnophila* batch cultures showed differences, the most prominent peaks were reproducible and would be the obvious candidates for structure elucidation. Once these prominent compounds have been determined, batch culture of *P. limnophila* may have to be further studied to enable isolation of the compounds which appear more variable in their production. It is possible that the life cycle switch of *P. limnophila* could cause fluctuations in the secondary metabolome (Jogler et al., 2011). *P. limnophila* daughter cells are flagellated and live as planktonic swimmers before developing into stalked sessile cells which are capable of dividing and forming biofilms (Jogler et al., 2011). If both cell types react to environmental cues in the same way remains unknown. The cultures used in our pilot study were not synchronized and thus the ratio between stalked sessile cells and flagellated swimmer cells might have varied among individual experiments. In addition, increased formation of cell aggregates might explain both the differences in production of secondary metabolites and the differences between OD_600_ and dry weight measurements. This is because aggregates give different OD_600_ values than single free-living cells. Despite such limitations, our approach will allow for the large scale production of planctomycetal compounds. For future studies, alternative reactor models and synchronization of cultures could increase reproducibility in the production of planctomycetal secondary metabolites.

In summary, we presented a pipeline for the utilization of Planctomycetes as sources of novel antibiotics. Screening of marine and limnic planctomycetal species demonstrated discovery of antimicrobial compounds. This finding further supports our hypothesis of allelopathic interactions between phototrophs and Planctomycetes, and emphasizes the usefulness of ecomimetic experiments to stimulate the production of bioactive molecules. This work will provide the starting point for the exploration of Planctomycetes as a currently untapped source of novel bioactive molecules.

## Author Contributions

OJ and CJ designed the project. OJ and MK cultivated all required bacteria with the help from MJ and extracted crude extracts from planctomycetal cultures. MK, OJ, and FS performed HPLC analysis. MK performed bioreactor experiments and measurements of biomass and glucose consumption. OJ performed with the help of MK activity assays. FS and BF performed HPLC fractionation and mass spectra analysis. PR did phylogenetic analysis. OJ and CJ wrote the manuscript with the help from FS and JW and input from all other authors.

## Conflict of Interest Statement

The authors declare that the research was conducted in the absence of any commercial or financial relationships that could be construed as a potential conflict of interest.

## Footnotes

1 https://www.dsmz.de/

2 https://www.dsmz.de/catalogues/dzif-sammlung-der-dsmz/indikatorstaemme-fuer-wirkstoffforschung.html.

## Supplementary Material

FIGURE S1 **Phylogenetic position of strain Pan216 within the phylum Planctomycetes**. The 16S rRNA gene-based tree was reconstructed under the maximum likelihood criterion with 1000 bootstrap resamplings and rooted to a cluster of sequences from anammox Planctomycetes (not shown). Strains of interest (isolate Pan216, *Planctopirus limnophila* and *Rhodopirellula baltica*) are highlighted in black and bold-faced. Validly published planctomycetal type strains are indicated by gray font and serve as matrix to pronounce the distinct phylogenetic position of strain Pan216. To estimate reliability of the tree branches, maximum parsimony and neighbor joining phylogenetic trees (not shown) were calculated (1000 bootstrap resamplings) and branch support was compared to the maximum likelihood phylogenetic tree. Black dots express bootstrap support values >70%, gray dots express support values >50% but <70%.

FIGURE S2 **High performance liquid chromatography analysis of the marine and limnic cultivation media for Planctomycetes. (A)** Full medium M1 for limnic Planctomycetes shows high signal peaks after a retention time from 3 to 10 min (intensity up to 8 × 10^5^ Au). **(B)** The chemically defined MM 1 shows no detectable signal peaks compared to its full medium M1. **(C)** Full medium M2 for marine Planctomycetes shows high signal peaks after retention time from 20 to 50 min (intensity up to 4 × 10^5^ Au). **(D)** The chemically defined MM2 shows no detectable signal peaks compared to M2.

FIGURE S3 **Dry weight determination of *P. limnophila* cells over 17 days of cultivation in 5 l fermentation with NAG**. The dry weight of *P. limnophila* cells increased from 0.6 g/l volume to 2.9 g/l volume after 17 days cultivation. The culture was fed after 7 days with 25 ml NAG and 10 ml vitamin solution (red arrow). This demonstrates that a similar dry-weight could be achieved with NAG as sole carbon source if a fed- batch procedure is employed.

FIGURE S4 **Reproducibility of the HPLC spectra of two distinct *P. limnophila* methanol extracts of 5 l fermentation**. In both HPLC spectra of the two distinct extracts similar signal peaks could be detected with slight shifts in retention time and intensity but also differences could be observed. Eight minor peaks (red asterisks) and one major peak (black asterisks) occurred only in one of the two replicates, while only the three major peaks (black arrows) were found in both extracts. The HPLC spectrum of the first extract (green curve) showed the first signal peak after, 7 min and a second after 15 min but no peak at 10 min while the HPLC spectrum of the second run (purple curve) showed the first peak at 10 but not after 7 or 15 min. The HPLC spectrum of the second extract revealed six signal peaks after a retention time of 25–32 min and an intensity of 1.56 × 10^5^ Au up to 10^6^ Au. In the first extract an additional signal peak at 24 min was detected, the intensity of the signal peak at 25 min decreased from 10^6^ Au to 9 × 10^5^ Au, while the peaks at 26 and 27 min were absent. A further additional signal peak in the HPLC spectrum of the first extract was detected at 52 min that was absent in the analysis of the second extract.

## References

[B1] Bengtsson M. M. Øvreås L. (2010). Planctomycetes dominate biofilms on surfaces of the kelp *Laminaria hyperborea*. *BMC Microbiol.* 10:261 10.1186/1471-2180-10-261 PMC2964680 20950420

[B2] Bengtsson M. M. Sjøtun K. Lanzen A. Øvreås L. (2012). Bacterial diversity in relation to secondary production and succession on surfaces of the kelp *Laminaria hyperborea*. *ISME J.* 6 2188–2198. 10.1038/ismej.2012.67 22763650 PMC3505018

[B3] Bengtsson M. M. Sjøtun K. Øvreås L. (2010). Seasonal dynamics of bacterial biofilms on the kelp *Laminaria hyperborea*. *Aquat. Microb. Ecol.* 60 71–83. 10.3354/ame01409

[B4] Berdy J. (2005). Bioactive microbial metabolites. *J. Antibiot.* 58 1–26. 10.1038/ja.2005.1 15813176

[B5] Christie-Oleza J. A. Piña-Villalonga J. M. Bosch R. Nogales B. Armengaud J. (2012). Comparative proteogenomics of twelve *Roseobacter* exoproteomes reveals different adaptive strategies among these marine bacteria. *Mol. Cell. Proteomics* 11 M111–013110. 10.1074/mcp.m111.013110 PMC3277765 22122883

[B6] Cooper M. A. Shlaes D. (2011). Fix the antibiotics pipeline. *Nature* 472:32 10.1038/472032a 21475175

[B7] Donadio S. Monciardini P. Sosio M. (2007). Polyketide synthases and nonribosomal peptide synthetases: the emerging view from bacterial genomics. *Nat. Prod. Rep.* 24 1073–1109. 10.1039/b514050c 17898898

[B8] Fowler T. Walker D. Davies S. C. (2014). The risk/benefit of predicting a post-antibiotic era: is the alarm working? *Ann. N. Y. Acad. Sci.* 1323 1–10. 10.1111/nyas.12399 24738913

[B9] Frank C. S. Langhammer P. Fuchs B. M. Harder J. (2011). Ammonium and attachment of *Rhodopirellula baltica*. *Arch. Microbiol.* 193 365–372. 10.1007/s00203-011-0681-1 21340506

[B10] Fuerst J. A. Sagulenko E. (2011). Beyond the bacterium: planctomycetes challenge our concepts of microbial structure and function. *Nat. Rev. Microbiol.* 9 403–413. 10.1038/nrmicro2578 21572457

[B11] Gade D. Stührmann T. Reinhardt R. Rabus R. (2005). Growth phase dependent regulation of protein composition in *Rhodopirellula baltica*. *Environ. Microbiol.* 7 1074–1084. 10.1111/j.1462-2920.2005.00784.x 16011746

[B12] Glöckner F. O. Kube M. Bauer M. Teeling H. Lombardot T. Ludwig W. (2003). Complete genome sequence of the marine planctomycete *Pirellula* sp. strain 1. *Proc. Natl. Acad. Sci. U.S.A.* 100 8298–8303. 10.1073/pnas.1431443100 12835416 PMC166223

[B13] Jeske O. Jogler M. Petersen J. Sikorski J. Jogler C. (2013). From genome mining to phenotypic microarrays: planctomycetes as source for novel bioactive molecules. *Antonie Van Leeuwenhoek* 104 551–567. 10.1007/s10482-013-0007-1 23982431

[B14] Jeske O. Schüler M. Schumann P. Schneider A. Boedeker C. Jogler M. (2015). Planctomycetes do possess a peptidoglycan cell wall. *Nat. Commun.* 6:7116 10.1038/ncomms8116 PMC4432640 25964217

[B15] Jogler C. Glöckner F. O. Kolter R. (2011). Characterization of *Planctomyces limnophilus* and development of genetic tools for its manipulation establish it as a model species for the phylum Planctomycetes. *Appl. Environ. Microbiol.* 77 5826–5829. 10.1128/AEM.05132-11 21724885 PMC3165242

[B16] Jogler C. Waldmann J. Huang X. Jogler M. Glöckner F. O. Mascher T. (2012). Identification of proteins likely to be involved in morphogenesis, cell division, and signal transduction in Planctomycetes by comparative genomics. *J. Bacteriol.* 194 6419–6430. 10.1128/JB.01325-12 23002222 PMC3497475

[B17] Jogler M. Jogler C. (2013). “Towards the development of genetic tools for Planctomycetes,” in *New Models for Cell Structure, Origins and Biology: Planctomycetes* ed. Fuerst J. A. (Heidelberg: Springer).

[B18] Lage O. M. Bondoso J. (2014). Planctomycetes and macroalgae, a striking association. *Front. Microbiol.* 5:267 10.3389/fmicb.2014.00267 PMC4042473 24917860

[B19] Lane D. J. (ed.) (1991). *16S/23S rRNA Sequencing.* Chichester: Wiley.

[B20] Lee K. C. Webb R. I. Fuerst J. A. (2009). The cell cycle of the planctomycete *Gemmata obscuriglobus* with respect to cell compartmentalization. *BMC Cell. Biol.* 10:4 10.1186/1471-2121-10-4 PMC2656463 19144151

[B21] Ludwig W. Strunk O. Westram R. Richter L. Meier H. Yadhukumar (2004). ARB: a software environment for sequence data. *Nucleic Acids Res.* 32 1363–1371. 10.1093/nar/gkh293 14985472 PMC390282

[B22] Morris R. M. Longnecker K. Giovannoni S. J. (2006). Pirellula and OM43 are among the dominant lineages identified in an Oregon coast diatom bloom. *Environ. Microbiol.* 8 1361–1370. 10.1111/j.1462-2920.2006.01029.x 16872400

[B23] Müller R. Wink J. (2014). Future potential for anti-infectives from bacteria - how to exploit biodiversity and genomic potential. *Int. J. Med. Microbiol.* 304 3–13. 10.1016/j.ijmm.2013.09.004 24119567

[B24] Pilhofer M. Rappl K. Eckl C. Bauer A. P. Ludwig W. Schleifer K. H. (2008). Characterization and evolution of cell division and cell wall synthesis genes in the bacterial phyla Verrucomicrobia, Lentisphaerae, Chlamydiae, and Planctomycetes and phylogenetic comparison with rRNA genes. *J. Bacteriol.* 190 3192–3202. 10.1128/JB.01797-07 18310338 PMC2347405

[B25] Pizzetti I. Fuchs B. M. Gerdts G. Wichels A. Wiltshire K. H. Amann R. (2011a). Temporal variability of coastal Planctomycetes clades at kabeltonne station, North Sea. *Appl. Environ. Microbiol.* 77 5009–5017. 10.1128/AEM.02931-10 21642408 PMC3147370

[B26] Pizzetti I. Gobet A. Fuchs B. M. Amann R. Fazi S. (2011b). Abundance and diversity of Planctomycetes in a Tyrrhenian coastal system of central Italy. *Aquat. Microb. Ecol.* 65 129–141. 10.3354/ame01535

[B27] Pruesse E. Peplies J. Glöckner F. O. (2012). SINA: accurate high-throughput multiple sequence alignment of ribosomal RNA genes. *Bioinformatics* 28 1823–1829. 10.1093/bioinformatics/bts252 22556368 PMC3389763

[B28] Strous M. Heijnen J. J. Kuenen J. G. Jetten M. S. M. (1998). The sequencing batch reactor as a powerful tool for the study of slowly growing anaerobic ammonium-oxidizing microorganisms. *Appl. Microbiol. Biotechnol.* 50 589–596. 10.1007/s002530051340

[B29] Tekniepe B. L. Schmidt J. M. Starr M. P. (1981). Life cycle of a budding and appendaged bacterium belonging to morphotype IV of the Blastocaulis-Planctomyces group. *Curr. Microbiol.* 5 1–6. 10.1007/BF01566588

[B30] van Niftrik L. Jetten M. S. (2012). Anaerobic ammonium-oxidizing bacteria: unique microorganisms with exceptional properties. *Microbiol. Mol. Biol. Rev.* 76 585–596. 10.1128/MMBR.05025-11 22933561 PMC3429623

[B31] van Teeseling M. C. Mesman R. J. Kuru E. Espaillat A. Cava F. Brun Y. V. (2015). Anammox Planctomycetes have a peptidoglycan cell wall. *Nat. Commun.* 6:6878 10.1038/ncomms7878 PMC4432595 25962786

[B32] Wegner C. E. Richter-Heitmann T. Klindworth A. Klockow C. Richter M. Achstetter T. (2012). Expression of sulfatases in *Rhodopirellula baltica* and the diversity of sulfatases in the genus *Rhodopirellula*. *Mar. Genomics* 9 51–61. 10.1016/j.margen.2012.12.001 23273849

[B33] Wilson M. C. Mori T. Ruckert C. Uria A. R. Helf M. J. Takada K. (2014). An environmental bacterial taxon with a large and distinct metabolic repertoire. *Nature* 506 58–62. 10.1038/nature12959 24476823

[B34] Zhao J. Yang N. Zeng R. (2008). Phylogenetic analysis of type I polyketide synthase and nonribosomal peptide synthetase genes in Antarctic sediment. *Extremophiles* 12 97–105. 10.1007/s00792-007-0107-9 17726573

