# Supplementary figures and images for: Developing Techniques for the Utilization of Planctomycetes As Producers of Bioactive Molecules
Source: Front Microbiol. 2016 Aug 19;7:1242. doi: 10.3389/fmicb.2016.01242 (PMC4990742; doi:10.3389/fmicb.2016.01242)

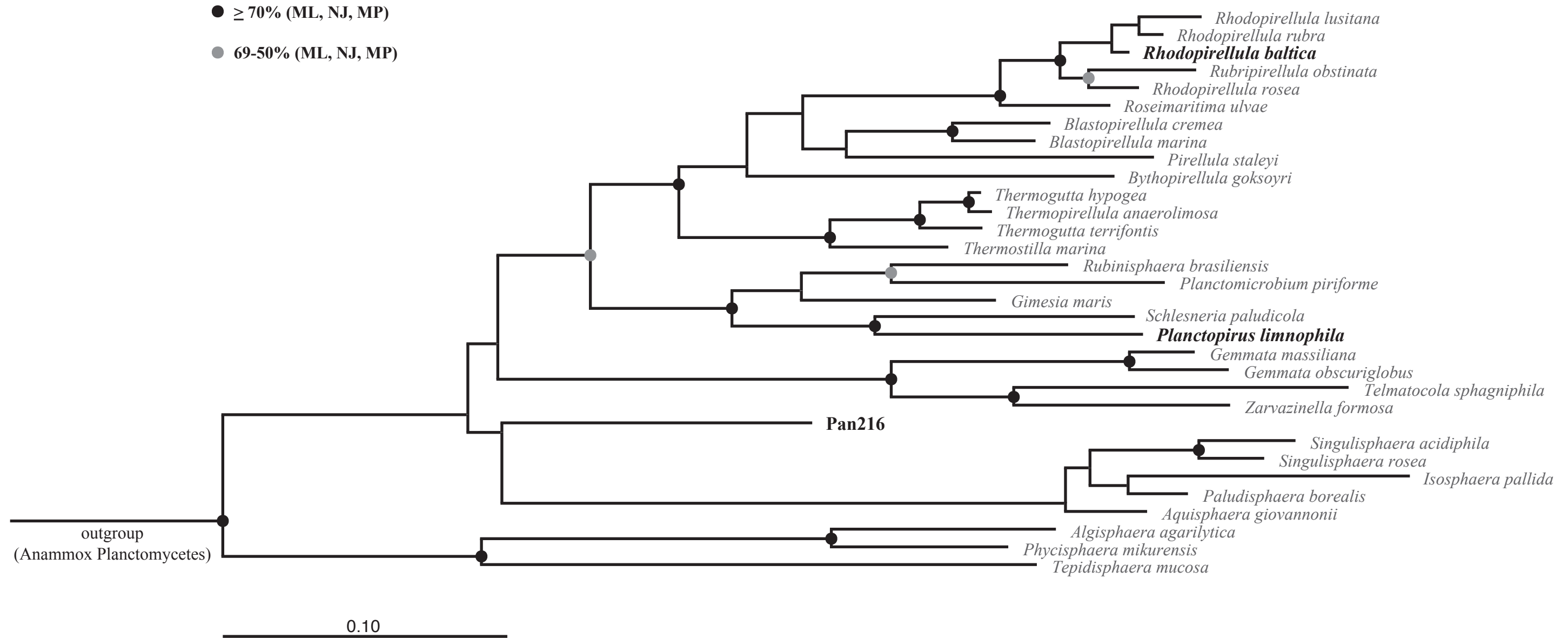

Supplement: FIGURE S1 — Phylogenetic position of strain Pan216 within the phylum Planctomycetes. The 16S rRNA gene-based tree was reconstructed under the maximum likelihood criterion with 1000 bootstrap resamplings and rooted to a cluster of sequences from anammox Planctomycetes (not shown). Strains of interest (isolate Pan216, Planctopirus limnophila and Rhodopirellula baltica) are highlighted in black and bold-faced. Validly published planctomycetal type strains are indicated by gray font and serve as matrix to pronounce the distinct phylogenetic position of strain Pan216. To estimate reliability of the tree branches, maximum parsimony and neighbor joining phylogenetic trees (not shown) were calculated (1000 bootstrap resamplings) and branch support was compared to the maximum likelihood phylogenetic tree. Black dots express bootstrap support values >70%, gray dots express support values >50% but <70%. [file Image_1.PDF]

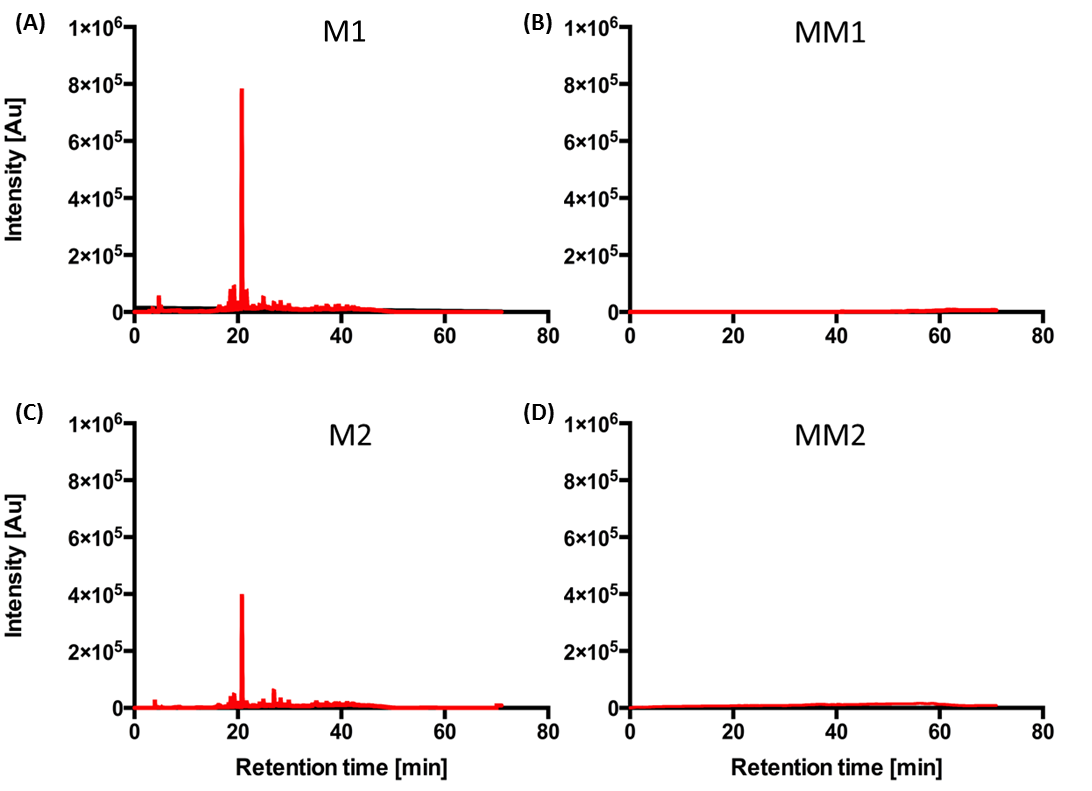

Supplement: FIGURE S2 — High performance liquid chromatography analysis of the marine and limnic cultivation media for Planctomycetes. (A) Full medium M1 for limnic Planctomycetes shows high signal peaks after a retention time from 3 to 10 min (intensity up to 8 × 10^5^ Au). (B) The chemically defined MM 1 shows no detectable signal peaks compared to its full medium M1. (C) Full medium M2 for marine Planctomycetes shows high signal peaks after retention time from 20 to 50 min (intensity up to 4 × 10^5^ Au). (D) The chemically defined MM2 shows no detectable signal peaks compared to M2. [file Image_2.TIF]

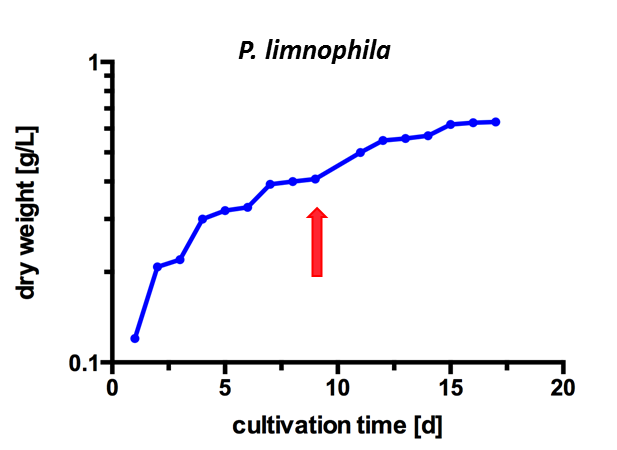

Supplement: FIGURE S3 — Dry weight determination of P. limnophila cells over 17 days of cultivation in 5 l fermentation with NAG. The dry weight of P. limnophila cells increased from 0.6 g/l volume to 2.9 g/l volume after 17 days cultivation. The culture was fed after 7 days with 25 ml NAG and 10 ml vitamin solution (red arrow). This demonstrates that a similar dry-weight could be achieved with NAG as sole carbon source if a fed- batch procedure is employed. [file Image_3.TIF]

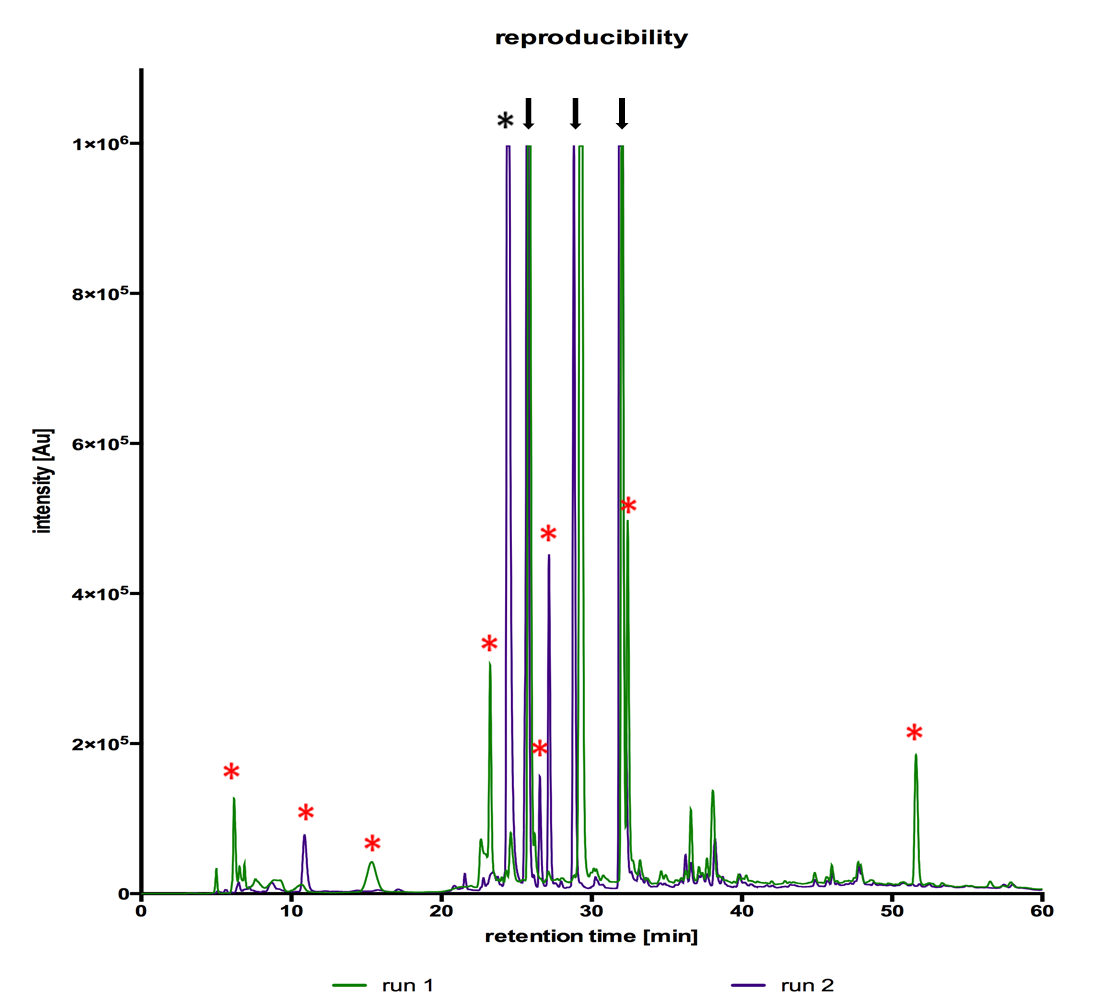

Supplement: FIGURE S4 — Reproducibility of the HPLC spectra of two distinct P. limnophila methanol extracts of 5 l fermentation. In both HPLC spectra of the two distinct extracts similar signal peaks could be detected with slight shifts in retention time and intensity but also differences could be observed. Eight minor peaks (red asterisks) and one major peak (black asterisks) occurred only in one of the two replicates, while only the three major peaks (black arrows) were found in both extracts. The HPLC spectrum of the first extract (green curve) showed the first signal peak after, 7 min and a second after 15 min but no peak at 10 min while the HPLC spectrum of the second run (purple curve) showed the first peak at 10 but not after 7 or 15 min. The HPLC spectrum of the second extract revealed six signal peaks after a retention time of 25–32 min and an intensity of 1.56 × 10^5^ Au up to 106 Au. In the first extract an additional signal peak at 24 min was detected, the intensity of the signal peak at 25 min decreased from 106 Au to 9 × 10^5^ Au, while the peaks at 26 and 27 min were absent. A further additional signal peak in the HPLC spectrum of the first extract was detected at 52 min that was absent in the analysis of the second extract. [file Image_4.TIF]
