# Supplementary material for: Developing Techniques for the Utilization of Planctomycetes As Producers of Bioactive Molecules
Source: Front Microbiol. 2016 Aug 19;7:1242. doi: 10.3389/fmicb.2016.01242 (PMC4990742; doi:10.3389/fmicb.2016.01242)
Supplement: Supplementary file 5 [file Data_Sheet_1.DOCX]

**Table S1:** **Sentinel strains for drug research of the DSMZ DZIF- collection (www.DSMZ.de)**

Used organisms for anti-microbial activity screening of planctomycetal extracts in minimum inhibitory concentration assays (MIC).

|  | ***B. subtilis***  **DSM10** | ***E.coli***  **DSM1116** | ***E.coli***  **TolC** | ***M. luteus***  **DSM1790** | ***S. aureus***  **DSM346** |
| --- | --- | --- | --- | --- | --- |
| **OD_600 of_ Aliquots** | 3.69 | 3.54 | 4.05 | 7.7 | 3.86 |
| **Amount cell suspension for 20 mL** | 54µl | 56µl | 49µl | 26µl | 52µl |
| **Temperature** | 30°C | 37°C | 37°C | 30°C | 30°C |
| **Start-OD_600_ in MIC-assay** | 0.01 | 0.01 | 0.01 | 0.01 | 0.01 |
| ***P. limnophila* inhibition in MIC-assay** | + | - | - | + | + |
| ***R. baltica* inhibition in MIC-assay** | + | + | + | + | + |
| **Pan216 inhibition in MIC-assay** | + | - | - | + | + |

**Table S2: List of reference strains used for 16S ribosomal RNA gene phylogenetic tree reconstruction.**

Strain designations and accession numbers were obtained from the NCBI database and the List of prokaryotic names with Standing in Nomenclature website (http://www.bacterio.net/-index.html).

| **Species** | **Strain** | **16S rRNA gene accession number** |
| --- | --- | --- |
| *Algisphaera agarylitca* | 06SJR6-2^T^ | AB845176 |
| *Aquisphaera giovannonii* | OJF2^T^ | NR_122081 |
| *Blastopirellula cremea* | Pr1d ^T^ | NR_118636 |
| *Blastopirellula marina* | LHWP2^T^ | NR_118153 |
| *Bythopirellula goksoyri* | SH 106^T^ | NR_029226 |
| *Gemmata massiliana* | IIL30^T^ | JX088244 |
| *Gemmata obscuriglobus* | UQM 2246^T^ | NR_114712 |
| *Gimesia maris* | 534-30^T^ | NR_025327 |
| *Isosphaera pallida* | IS1B^T^ | NR_074534 |
| *Paludisphaera borealis* | PX4^T^ | KF467528 |
| *Phycisphaera mikurensis* | FYK2301M01^T^ | NR_074491 |
| *Pirellula staleyi* | ATCC 27377^T^ | NR_074521 |
| *Planctomicrobium piriforme* | P3^T^ | KP161655 |
| *Planctopirus limnophila* | Mü 290^T^ | NR_074670 |
| *Rhodopirellula baltica* | SH 1^T^ | NR_043384 |
| *Rhodopirellula lusitana* | UC17^T^ | EF589351 |
| *Rhodopirellula rosea* | LHWP3^T^ | JF748734 |
| *Rhodopirellula rubra* | LF2^T^ | HQ845500 |
| *Roseimaritima ulvae* | UC8^T^ | HQ845508 |
| *Rubinisphaera brasiliensis* | DSM 5305^T^ | NR_074297 |
| *Rubripirellula obstinata* | LF1^T^ | DQ986201 |
| *Schlesneria paludicola* | MPL7^T^ | NR_042466 |
| *Singulisphaera acidiphila* | MOB10^T^ | NR_102439 |
| *Singulisphaera rosea* | S26^T^ | NR_116969 |
| *Telmatocola sphagniphila* | SP2^T^ | NR_118328 |
| *Tepidisphaera mucosa* | 2842^T^ | KM036168 |
| *Thermogutta hypogea* | SBP2^T^ | KC867695 |
| *Thermogutta terrifontis* | R1^T^ | KC867694 |
| *Thermopirellula anaerolimosa* | VM20-7^T^ | AB558583 |
| *Thermostilla marina* | SVX8^T^ | KR872395 |
| *Zarvazinella formosa* | A10^T^ | NR_042465 |
| *Ca.* Brocadia anammoxidans | - | AF375994 |
| *Ca.* Brocardia sinica | JPN1 | AB565477 |
| *Ca.* Jettenia asiatica | AS-1 | DQ301513 |
| *Ca.* Kuenenia stuttgartiensis | - | CT573071 |
| *Ca.* Scalindua wagneri | - | EU478692 |
| *Ca.* Scalindua brodae | - | AY257181 |
